# Development and refinement of the sore throat pain model as an assay for measuring therapeutic effects on acute pain

**DOI:** 10.3389/fpain.2025.1576168

**Published:** 2025-06-23

**Authors:** Bernard P. Schachtel, Adrian Shephard

**Affiliations:** ^1^ Yale School of Public Health, New Haven, CT, United States; ^2^ Schachtel Research Company, Inc, Jupiter, FL, United States; ^3^ Reckitt Benckiser Healthcare International Ltd, Slough, United Kingdom

**Keywords:** acute pain, sore throat, pain model, pharyngitis, methodology, rating scales, treatment effects, symptoms

## Abstract

The physical and subjective status of patients with acute throat pain has been developed and refined over the past 40 years as an acute pain model to measure changes in patient-reported symptoms attributed to active pharmacologic intervention when patients with painful pharyngitis are evaluated under randomized, double-blind, placebo-controlled conditions. Acute, painful pharyngitis is a familiar experience for the majority of adults and children (“a sore throat” is the most common example of the aches and pains of the common cold). As such, the condition has served as a general acute pain model to demonstrate the acute effects of non-prescription-strength analgesic agents (for mild-to-moderate pain) and prescription-strength analgesics (for moderate-to-severe pain). Here we discuss the methodologic features of this clinical pharmacology assay as it was refined from its original examinations of classic, orally administered, acute analgesics (aspirin, acetaminophen, aspirin with caffeine, ibuprofen) to its more recent evaluations of celecoxib, valdecoxib, topical benzydamine, and topical flurbiprofen.

## Introduction

Painful pharyngitis is a common experience of all people from childhood to adulthood (1, 2). Indeed, a “sore throat” was aptly described by Jane Austen as “worse than anybody else's” (3), underscoring both the commonality and variability of the condition (i.e., no two sore throats are “exactly” the same for an individual or among different individuals). Over a 12-month period, approximately half of adults experience at least one episode of throat discomfort (4), and most people suffer from pharyngitis many times throughout their lives (5) with incidence and prevalence being greater in childhood and early adulthood (6). Pharyngitis remains one of the most common reasons for consultations with healthcare services (7), exerting physical and emotional impact and significantly affecting school and work attendance and general quality of life (4, 8, 9). Overall, in many ways a sore throat is the most common type of acute pain.

Viral upper respiratory tract infections (URTIs) are responsible for the majority of sore throats (10–12), although bacterial infections and environmental factors account for a small proportion of cases (13, 14). While several bacterial species have been implicated in the etiology of pharyngitis, group A b-hemolytic streptococcus (GABHS) and group C b-hemolytic streptococcus (GCBHS) are the most common bacterial etiologies of pharyngitis. Microbiologically identified cases of GABHS are treated with antibiotics to prevent complications such as acute rheumatic fever and possibly reduce pharyngeal symptoms; cases of GCBHS are not treated with antibiotics unless complications are suspected (as from prolonged symptomatology) (10, 15).

Regardless of the etiology, inflammation of the oropharynx is responsible for multiple patient-reported symptoms (13, 16). The physical signs of oropharyngeal inflammation are directly observable on patient examination (17, 18). The production of inflammatory signs and symptoms has provided an opportunity for the development of targeted treatments for sore throat, including a variety of topical and systemic medications. While doctors had historically been advising patients with a sore throat due to a cold to “take two aspirin” (19), this recommendation was made in the absence of any published pharmacologic evidence that aspirin (acetylsalicylic acid), a systemic non-steroidal anti-inflammatory drug (NSAID), was effective compared to placebo for this indication.

A standardized, validated clinical pharmacologic assay was needed to overcome methodologic challenges that had heretofore obfuscated the clinical observation that aspirin, “the gold standard” of non-prescription-strength analgesics at the time, was indeed effective for the acute pain of tonsillopharyngitis, as observed by clinicians and their patients. The 1984 study report met this criterion, demonstrating that the “positive control” aspirin was effective compared to placebo under double-blind, randomized study conditions. Thus the model itself was validated by the evidence of aspirin efficacy compared to placebo (20).

The first publication of a randomized controlled trial for studying painful pharyngitis appeared in Clinical Pharmacology & Therapeutics in 1984 (20). This design, now known as the sore throat or pharyngitis pain model, has since been refined for the evaluation of other pharmacologic agents, including ibuprofen, aspirin with caffeine, ketoprofen, celecoxib, parecoxib, topical benzydamine, and, most recently, topical flurbiprofen (19–31). This review of the development and refinement of the sore throat pain model over the past 40 years examines the fundamental principles of clinical trial design employed in this clinical pharmacology assay, emphasizing (a) quantitative patient-directed outcome measures which are appropriate to (b) the defined medical condition, including (c) pertinent confounding clinical features, each of which contributes to its sensitivity as an assay of therapeutic response.

## Core features of the sore throat pain model

Developed as a pharmacologic assay to assess the efficacy of analgesic agents (18–20, 31), the sore throat pain model follows the fundamental principles of clinical trial design (32–34), including confirmation of the clinical condition, the use of sensitive rating scales, uniformity in the pre-treatment status of subjects and elimination of clinical confounding variables (35). The initial clinical trial established the basic study design features required to demonstrate under double-blind, placebo-controlled conditions the efficacy and safety of single-dose, low-dose analgesics, in this case, aspirin (650 mg) and acetaminophen (650 mg) compared with placebo in patients with pharyngitis (20).

## Confirmation of a defined clinical condition and uniformity in pre-treatment status of patients with painful pharyngitis

When considering the design of a model to assess treatments for sore throat, it is essential to ensure the homogeneity of subjects in each treatment group at baseline. Foremost among admission criteria for the sore throat pain model was the requirement that patients had clinically observed pharyngitis (18, 19, 30, 31). Infectious and non-infectious factors can trigger the production of pro-inflammatory mediators (13, 16), which initiate an inflammatory cascade ultimately resulting in the four cardinal signs of inflammation—pain, swelling, heat, and redness (16, 36, 37). It is these cardinal signs that are detected on physical examination of patients with a sore throat. In the initial implementation of the model, therefore, five objective findings were selected which are indicative of oropharyngeal inflammation (oral temperature, oropharyngeal color, oropharyngeal enanthemas, cervical adenopathy, and cervical adenitis), with each feature being rated on a familiar 0–2 ordinal scale by the examining clinician. These findings were summarized in an 11-point index called the tonsillopharyngitis score, or TPS (18). This objective assessment of the clinical condition thus specified and confirmed the presence of one pain-producing condition (i.e., pharyngitis), with physical examination excluding other local painful conditions (e.g., laryngitis, pharyngeal abscess), and ensured that all subjects revealed the de minimis physical findings to establish the diagnosis of pharyngitis.

## Elimination of clinical confounding variables in patients with URTI and painful pharyngitis

A further pre-treatment criterion of the model was based solely on a clinical observation: patients with pharyngitis as an expression of URTI often have other manifestations of URTI, in particular, nasal congestion and cough. Because severe nasal congestion (“a stuffy nose”) can obstruct the nares, causing mouth-breathing which can dry the throat (and thus worsen a sore throat), all patients in the initial clinical trial were evaluated for nasal congestion on an ordinal scale, the Nasal Congestion Assessment (NCA) (18). Approximately one-third of the 150 otherwise qualified patients with sore throat due to pharyngitis had mouth-breathing due to nasal congestion: their results were shown to confound analgesic responses and obscure the differentiation of known active drugs from placebo (18). For this methodologic reason, in all subsequent trials using this pain model, patients with mouth-breathing were excluded from entry. Similarly, from a common-sense perspective, it was reasoned that coughing that causes discomfort to the back of the throat can physically worsen throat pain in subjects with pharyngeal inflammation. These patients were excluded from entering the clinical trial (lest a disproportionate number of subjects with cough could be randomly assigned to an active treatment group, confounding true pharmacologic effects).

## Use of sensitive rating scales for patients with painful pharyngitis

Another feature of this pain model was the structure of the measurement instruments for pain intensity and pain relief. The invention of linear or visual analog scales (VAS) for pain assessment by British psychologists (38) provided a potentially more sensitive instrument to measure pain intensity than the then-conventional categorical pain intensity scale, allowing patients greater opportunity to express (numerically) the intensity of pain and readily convey changes in intensity over time after treatment. The initial implementation of the sore throat pain model therefore required patients at screening to circle the category (mild, moderate, severe) that best described their throat pain, or odynophagia, on a categorical pain scale and to indicate pain intensity on a 100 mm horizontal VAS (with endpoints of no pain and severe pain): this VAS is known as the Sore Throat Pain Intensity Scale [STPIS] (18). Scores of 50–74 mm and 75–100 mm on the STPIS (indicating moderate and severe pain, respectively) were compared to each subject's categorical pain scale selection (mild, moderate, or severe). Excellent agreement was identified between the degree of patient-reported baseline throat pain measured on the STPIS and pain intensity measured on the categorical pain scale. Interestingly, there was also a strong correlation between the index of objective evidence of pharyngeal inflammation (on the clinician-assessed TPS) and subjective response to pharyngeal inflammation (on the patient-reported STPIS) (18, 20).

As required for other acute pain models, a pre-treatment criterion of the model specified that only patients with at least moderate pain intensity on the categorical pain scale were eligible for admission. Thus, only subjects with moderate or severe pain were admitted to the clinical trial. To ensure that patients with moderate or severe pain were represented equally in each treatment group, patients were randomized to treatment within each pain intensity stratification. In line with observations by previous clinical investigators and the common-sense principle in physics that higher levels of a condition facilitate the detection of change in the condition (i.e., greater differences are more likely detected from greater initial states), examination of post-treatment responses for all patients and for each stratum of pain intensity demonstrated a significant relationship between pre-treatment pain level and response to treatment (20). Higher levels of pre-treatment pain do, in fact, facilitate differentiation between active drug and placebo.

To enable patients to record greater expression of relief after treatment, the initial implementation of the model utilized the linear STPIS and a 5-category relief scale (no relief, mild relief, moderate relief, almost complete relief, complete relief) rather than the then-conventional 4-category relief scale. (Outcomes measured on this 5-category relief scale demonstrated significant differentiation between each active drug and placebo.) Another new measurement instrument was employed in the initial study, a transitional scale that permitted the subject to directly compare each current pain intensity level to his/her previous level of pain intensity (rather than rely on repeated post-study arithmetic subtractions of the current pain intensity level from the pre-treatment level, summed to relate “pain intensity difference”). The subject used a 100 mm VAS—a change-in-pain scale—with “much worse” at the left end, “same” in the middle, and “much better” at the right end in an effort to discern, if possible, greater differentiation of active from placebo in the trial (18). Results on this transitional scale agreed with computed summed pain intensity differences, confirming not just the utility of a bi-directional, or transitional, VAS to relate changes in pain intensity but also providing confirmatory evidence of the efficacy of aspirin and acetaminophen compared to placebo in this assay.

Using the above criteria, clinical status indices, and assessment scales, the initial implementation of the sore throat pain model confirmed that aspirin (650 mg) and acetaminophen (650 mg) were significantly more effective than placebo, indicating the sensitivity of the sore throat pain model as a clinical pharmacology assay of acute analgesic activity and confirming clinicians’ advice to use these agents for sore throat pain (18). The upside assay sensitivity of the sore throat pain model has been substantiated further through demonstration of a dose-dependent relationship of aspirin 500 mg and 1,000 mg (39).

## Refining the clinical evaluation of tonsillopharyngitis

To provide clinicians with greater opportunity to report their findings on physical examination, the TPS was expanded into a 21-point index based on seven clinical findings (oral temperature, oropharyngeal color, number of oropharyngeal enanthems, largest size of anterior cervical lymph nodes, number of anterior cervical lymph nodes, maximum tenderness of anterior cervical lymph nodes, and tonsillar size), called the tonsillopharyngitis assessment, or TPA, in which each feature is rated on a commonly used 0–3 ordinal scale (Table 1) (18, 30).

**Table 1:** Full details of the tonsillopharyngitis assessment (TPA), reproduced from (27).

Finding	0 points	1 point	2 points	3 points
Oral temperature	≤98.6°F	98.7–98.9°F	99.0–99.9°F	≥100°F
Oropharyngeal color	Normal/pink	Slightly red	Red	Beefy red
Size of tonsils	Normal/absent	Slightly enlarged	Moderately enlarged	Much enlarged
Number of oropharyngeal enanthems (vesicles, petechiae or exudates)	None	Few	Several	Many
Largest size of anterior cervical lymph nodes	Normal	Slightly enlarged	Moderately enlarged	Much enlarged
Number of anterior cervical lymph nodes	Normal	Slightly increased	Moderately increased	Greatly increased
Maximum tenderness of some anterior cervical lymph nodes	Not tender	Slightly tender	Moderately tender	Very tender

The TPS and TPA are highly correlated (*r* = 0.807; *p* ≤ 0.001), with the refinements of the TPA enabling a more representative clinical assessment of the physical signs of tonsillopharyngitis (35). However, at the upper extreme, the TPA does not seem to specify patients complaining of “a bad sore throat”; while they rate their symptoms (pain, swollen throat, difficulty swallowing) as severe (i.e., greater than 90 mm on 100 mm VAS), physical findings on the TPA only modestly agree with this degree of symptomatic severity (in one study, for example, the mean TPA was 10.4 on this 21-point index, not in the upper third of index scores as might be expected (40).

In this type of research, physical features such as tonsillar exudates and anterior cervical lymphadenopathy were specifically examined, as focused upon in the Centor criteria used to predict the likelihood of positive Strep A culture in a given patient (17). Utilizing the TPA and measurements of individual throat symptoms as well as sore throat pain intensity, this study revealed that two features of the TPA (at least moderately enlarged tonsils, at least moderately tender anterior cervical lymph nodes), as well as the overall TPA, helped differentiate between bacterial and viral etiologies of sore throat. However, the sensitivities and specificities of these specific clinical indicators were modest (Table 2) (41).

**Table 2:** Association between Strep A throat culture and objective and subjective features of pharyngitis, adapted from (41).

Features	*p*-value for Strep A vs non-Strep A	Sensitivity, %	Specificity, %
Objective features			
TPA ≥ median (9)	<0.01	75.0	52.4
Oral temperature ≥99°F	0.1221	37.5	74.0
Oropharyngeal color at least red	0.3447	77.5	29.6
Tonsils at least moderately enlarged	<0.001	72.5	56.2
At least several oropharyngeal enanthems	0.6409	20.0	76.7
Anterior cervical lymph nodes at least moderately enlarged	0.2848	47.5	61.2
Anterior cervical lymph nodes at least moderately increased	0.9288	22.5	78.1
Anterior cervical lymph nodes at least moderately tender	<0.05	62.5	57.3
Subjective features			
Pain (STPIS ≥ median [79 mm)	0.0743	65.0	49.9
Difficulty swallowing (DSS ≥ median [78 mm)	0.5821	55.0	49.6
Swollen throat (SwoTS ≥ median [78 mm)	<0.05	70.0	48.8

DSS, difficulty swallowing scale; TPA, tonsillopharyngitis assessment; STPIS, sore throat pain intensity scale; SwoTS, swollen throat scale.

## Refining the model focusing on patient-reported descriptors of pharyngitis

As in the treatment (and the assessment of treatments) of other painful conditions, to relieve painful pharyngitis it is important that treatments meet the specified symptomatic needs and expectations of the patient. Like other clinical conditions with patient discomfort, each patient's experience and perception of sore throat is highly variable (42). For patients with pharyngitis these symptoms extend beyond the evaluation of the severity of pain (an evaluative dimension) to other sensory and affective (emotional) dimensions of pain (43) and functional outcomes. To enhance the capability of the sore throat pain model to measure these different, condition-specific symptoms and their change, or not, after treatment, actual patient-reported symptoms were identified from a survey of 150 adults complaining of a sore throat. In this research, patients were asked to describe their sore throat in their own words and to complete the sentence “My throat hurts so much that…” also using their own words (18). Not surprisingly, as clinicians have encountered in practice, most patients volunteered descriptors in common parlance such as “it feels scratchy”, “it's difficult to swallow”, “my throat is swollen”, “it hurts”, etc. While many of these descriptors were evaluative, referring to pain intensity (e.g., sore), many other words were sensory (e.g., burning/hot), others were affective (e.g., annoying), and, critically, some were functional (e.g., difficulty swallowing, difficulty talking) (18). In designing later versions of the sore throat pain model, therefore, these truly “patient-reported” throat-related symptoms were developed as measurement instruments and endpoints.

## Incorporating sensory, affective, and functional descriptors of pain used by patients with painful pharyngitis

**Figure 1:**
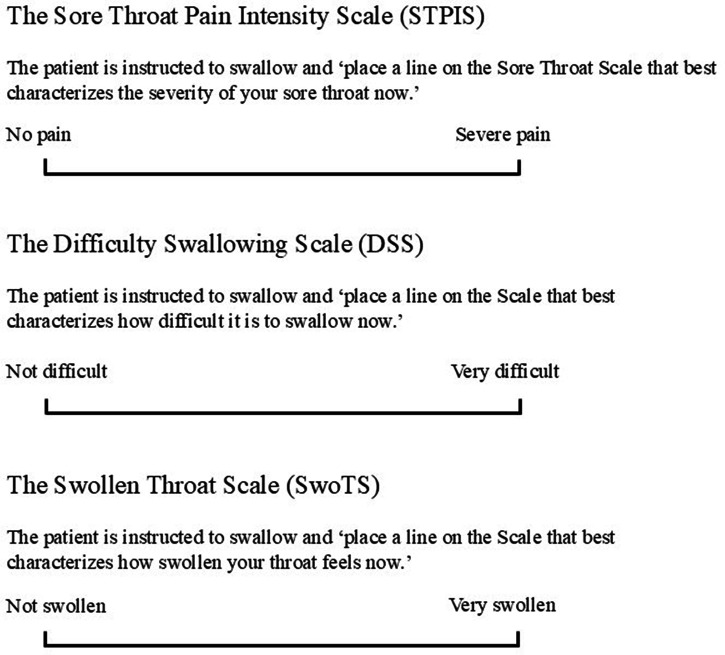
Visual analog scales (100 mm) to assess patient reported outcomes. **(A)** To assess sore throat pain, **(B)** to assess difficulty swallowing and **(C)** to assess sensation of swollen throat, reproduced from (27).

Difficulty swallowing (dysphagia) and the sensation of a swollen throat were the most common patient-reported throat descriptors identified in the survey (18). These terms were therefore measured in specific 100 mm VAS [subsequently known as the Difficulty Swallowing Scale, or DSS, and the Swollen Throat Scale, or SwoTS (31)]. In a study comparing ibuprofen (400 mg), acetaminophen (1,000 mg), and placebo, throat pain was assessed using the STPIS, while difficulty swallowing and swollen throat were also evaluated (on the DSS and the SwoTS, respectively) (Figure 1) (31).

All three scales in this 1988 study were sensitive, discriminating between each active medicine and placebo and detecting differences between active agents on all three scales (all *p* < 0.01) (31). The same “quality-of-pain” scales were also used in a study investigating the use of aspirin 800 mg with caffeine 64 mg compared with aspirin 800 mg and placebo for the treatment of sore throat (25). Aspirin with caffeine was found to be significantly more effective than aspirin alone on the pain intensity scale, on the DSS, and on the SwoTS (all *p* < 0.05), demonstrating that caffeine is an analgesic adjuvant with aspirin. From a methodologic perspective, these newer rating scales were validated by producing the same results as the standard pain intensity scale (25).

Even so, other research on patients with sore throat revealed that throat pain, the sensation of a swollen throat and difficulty swallowing are independent patient-reported outcomes (PROs) (42). While these three PROs are related, they are not identical (with Spearman rank correlation coefficients ranging from *r* = 0.54–0.80), with evidence of individual, variable, and independent ratings on each PRO. In most patients (∼81%) at least one of these three pharyngeal symptoms was assessed with a distinctly different intensity (i.e., at least 10 mm higher rating on the specific 100 mm VAS) from another (42). As such, these different PROs can be utilized as different therapeutic endpoints in analgesic clinical trials.

## Identifying the chief complaint of patients with painful pharyngitis

In many clinical studies on symptomatic disease, investigators select a standard PRO as the primary endpoint. However, as already described, patients with painful pharyngitis experience a range of independent symptoms (e.g., throat pain, swollen throat, and difficulty swallowing), which they articulate in a variety of ways when describing their condition (2, 42, 44). While “pain” may globally describe the patient's discomfort, the conventional or customary primary endpoint selected by the investigator or drug regulator, pain, may not be the specific symptom that bothers the patient most. Consequently, from the clinical investigator's perspective, pain intensity may not be the optimal or most efficient endpoint to use to detect change over time attributable to investigational treatment.

A further refinement of the patient-centric methodology of the sore throat pain model, therefore, was consideration of the patient's “chief complaint”—i.e., the most bothersome symptom from the patient's perspective (and the main focus of the clinician's evaluation of a patient while taking a medical history, in fact). In other research, patients’ ratings of each pharyngeal symptom were ranked to designate which feature signified their chief complaint (45). Analyses were conducted according to the patients’ chief complaint. Patients whose chief complaint was sore throat pain, for example, experienced significantly greater pain reduction with flurbiprofen 8.75 mg lozenges compared with placebo lozenges (*p* < 0.0001). Not surprisingly, patients whose chief complaint was “pain” registered greater drug effect on throat pain than patients whose chief complaint was “a swollen throat” or “difficulty swallowing” (similar, but not as dramatic, evidence of drug activity compared to placebo was shown for patients when they used their own “chief complaint” scale, both *p* < 0.05) (45). As has been subsequently shown in studies on patients with migraine (46, 47), these results indicate the enhanced assay sensitivity of using the patient's own chief complaint (i.e., his/her “most bothersome symptom”) as an endpoint in analgesic trials (45).

## Adding throat soreness as an evaluative descriptor for patients with painful pharyngitis

In his seminal research on the study of pain and its treatment Lasagna created a “pain thermometer” to measure pain intensity (48). This vertical 11-point ordinal scale was expanded into a demarcated 21-point (20 cm) vertical ordinal scale (the Sore Throat Pain Thermometer [STPT) for the evaluations of liquid formulations of benzydamine and of ibuprofen in children with sore throat, demonstrating significant differences between each active drug and placebo (49, 50). In the study comparing benzydamine suspension to placebo, pain was rated by the child using both the STPT and a five-category smiley face Children's Sore Throat Relief Scale [patterned after the work of Rogers (51)], as well as being assessed independently by the research nurse (49). Benzydamine was significantly more effective than placebo (*p* < 0.05) by all three measures (confirming not only the blunt “honesty” of children as research patients but also the clinical acumen of nurse observers). In the study comparing ibuprofen, acetaminophen, and placebo (50), children used the STPT to assess their sore throat pain intensity and the five-face Children's Sore Throat Relief Rating Scale to assess their pain relief. Both active treatments were found to be significantly more effective (*p* < 0.05) compared with placebo on these children's rating scales (findings that were also demonstrated independently in the office by their pediatricians and mothers, both reliable observers of children's discomfort and their response to treatment), confirming that the sore throat pain model (and, in particular, Lasagna's vertical ordinal pain intensity scale) can be used in children to distinguish active treatment from placebo under controlled conditions.

As “sore” is an evaluative term that patients commonly use to describe the quality of pain associated with pharyngitis, a later refinement of the model incorporated this patient-reported word to evaluate throat soreness directly (35). Based on Lasagna's vertical pain thermometer (48), a vertical 11-point throat soreness scale (generically named the Lasagna Pain Scale, or LPS, after its inventor) was included in a randomized controlled trial demonstrating the efficacy of the cyclooxygenase-2 (COX-2) selective inhibitor valdecoxib compared to placebo for the relief of sore throat (35). Good correlation was noted between post-treatment scores on the LPS and those from a conventional visual analog pain intensity scale (*r* = 0.792–0.973, *p* ≤ 0.001), providing validation of the LPS as a sensitive instrument measuring throat soreness. The LPS was also used in a proof-of-concept study examining the adjuvant effect of an H1-antagonist (hydroxyzine) and of an H2-antagonist (nizatidine) when added to ibuprofen. Summed pain intensity differences measured over 6 hours on the LPS revealed significantly greater pain reduction for each histamine antagonist compared to ibuprofen (both *p* < 0.05) (52), marking another use of the sore throat pain model to demonstrate analgesic potentiation and signifying the utility of a vertical 11-point ordinal pain intensity scale.

## Determining the definite improvement level of patients with painful pharyngitis

It is of course important to appreciate that a statistical improvement does not necessarily translate to an improvement that is meaningful for the patient. Therefore, efforts were made to account for the patient's own definition of improvement within the context of a clinical trial. We had observed that many patients would report to their clinicians that they were “definitely improved” by a medication prescribed for their condition. This real-life endpoint prompted a further refinement to the study model, namely, asking each patient within a clinical trial setting to identify his/her own “definite improvement level”, or DIL, for assessments of pain, difficulty swallowing, and the sensation of a swollen throat (53). This “patient-determined” method was used to evaluate the efficacy of flurbiprofen 8.75 mg lozenges. At baseline, patients rated their throat symptoms on three different 100 mm VAS scales: the STPIS, DSS, and SwoTS. At the conclusion of the trial, after all trial assessments had been completed before discharge from the study, each patient was asked to indicate (on a copy of his/her completed baseline rating scale) which rating in symptom severity would represent “definite improvement” relative to this baseline rating. The patient-reported definite improvement levels varied widely for all three scales; analyses based on each patient's DIL, however, identified significant differences between the flurbiprofen and placebo treatment groups (*p* < 0.05). Interestingly, when comparing percentage changes in pain intensity based on each patient's DIL and the pain intensity difference of the treatment group, 55% pain intensity difference was indicative of definite improvement, which is analogous to the classic “pain half-gone” criterion of efficacy used as a definitive endpoint by early analgesiologists (54). The DIL, we conclude, is a reliable and very “patient-centered” endpoint that can be used to determine analgesic efficacy.

## Determining meaningful pain relief in patients with painful pharyngitis

Because the patient's report of at least moderate pain relief has been identified as an indicator of definite and clinically meaningful pharmacologic activity (55), research was conducted to determine the percentage of patients with sore throat who reported at least moderate relief following use of flurbiprofen 8.75 mg or placebo lozenge (28, 56). Relief was reported at prespecified timepoints on a 6-category pain relief scale (no relief, slight relief, mild relief, moderate relief, considerable relief, complete relief). By this metric it was shown that significantly more flurbiprofen-treated patients achieved at least moderate relief compared to placebo (*p* < 0.001). This finding confirmed results from the conventional summed pain intensity analyses performed in this study while validating the criterion of “moderate relief” as a definitive standard for demonstrating drug action (28). Using this methodology, we thus learned that the sore throat pain model can be employed to demonstrate, if present, clinically meaningful pharmacologic activity.

## Developing a composite measure for a sore throat: the qualities of sore throat Index (QuaSTI)

**Figure 2:**
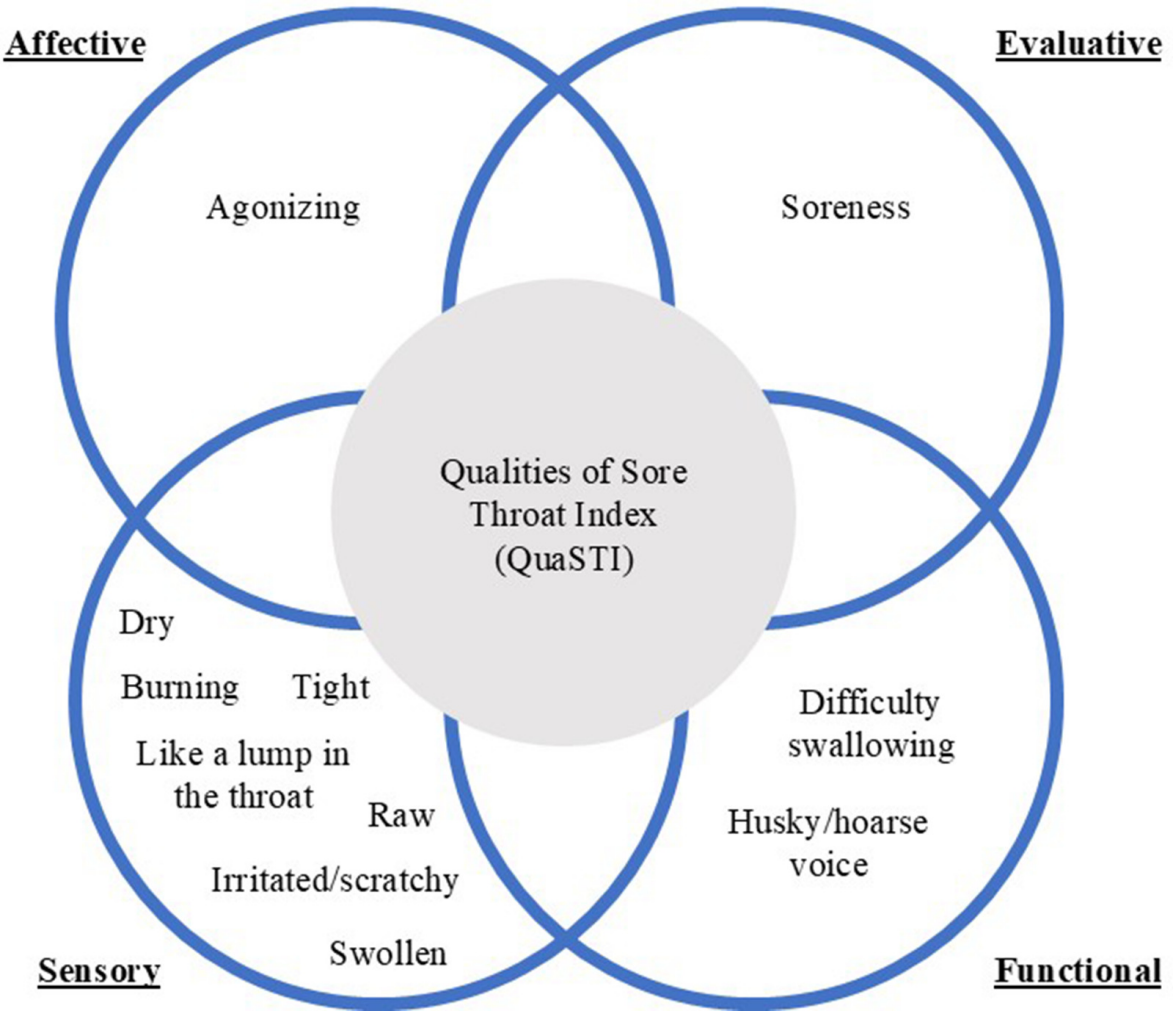
Qualities of Sore Throat Index (QuaSTI). Schematic presentation of words and phrases used to describe sensory, functional, affective, and evaluative pain qualities of sore throat symptoms.

The predominant sensory, evaluative, functional, and affective qualities of pain commonly described by patients were later combined with the assessment of soreness on the LPS into a composite indicator of the status of a patient with sore throat, the QuaSTI. This measurement instrument comprises eleven 0–10 Likert scales: seven sensory throat symptoms (burning, raw, dry, irritated/scratchy, tight, like a lump in the throat, swollen), two functional throat symptoms (husky/hoarse voice, difficulty swallowing), one affective throat descriptor (agonizing), and one evaluative quality (soreness) (44). Further research on the QuaSTI identified and quantified how assessments of these symptoms clustered (showing differentiation and overlapping), as shown in Figure 2 (44, 57, 58).

Change in the QuaSTI was examined as a therapeutic endpoint in a study comparing flurbiprofen 8.75 mg lozenge with placebo. Mean change in the QuaSTI score was significantly greater in the flurbiprofen treatment group compared with the placebo group (*p* < 0.05). The QuaSTI proved to be a sensitive measure for evaluating analgesic efficacy in patients with sore throat, demonstrating significant improvements in all truly “patient-articulated” terms (57).

## Determining the onset of pain relief in patients with painful pharyngitis

Researchers have demonstrated correlation between the onset of acute pain relief and better overall pain relief, a finding which may explain why patients desire fast-acting medicines (59). A variety of methods have been used to study onset of action in analgesic trials on patients with sore throat (28). Assessing pain at frequent timepoints (e.g., at 5- or 10-minute intervals over the first hour) represents one such method (29, 60–62). A randomized, double-blind, placebo-controlled trial in patients with sore throat used 5-minute intervals between assessments of pain intensity over the first 60 minutes after dosing with 200 and 400 mg doses of ibuprofen (62). This methodology enabled patients to report a significant (*p* < 0.05) effect of ibuprofen 400 mg on pain intensity compared to placebo beginning at 20 minutes post dose. The 5-minute interval methodology was used in another randomized, double-blind, placebo-controlled study investigating the pharmacodynamic profile of a tablet formulation of acetaminophen 500 mg combined with sodium bicarbonate 630 mg. Here, too, because patients were asked frequently about their symptoms at early timepoints, they were able to report a significant (*p* ≤ 0.03) difference in sore throat pain relief between the new acetaminophen formulation and placebo beginning at the 15-minute timepoint (60). Notably, in neither study was an enhanced and/or early placebo response detected which could confound the reporting of early pharmacologic activity.

**Figure 3:**
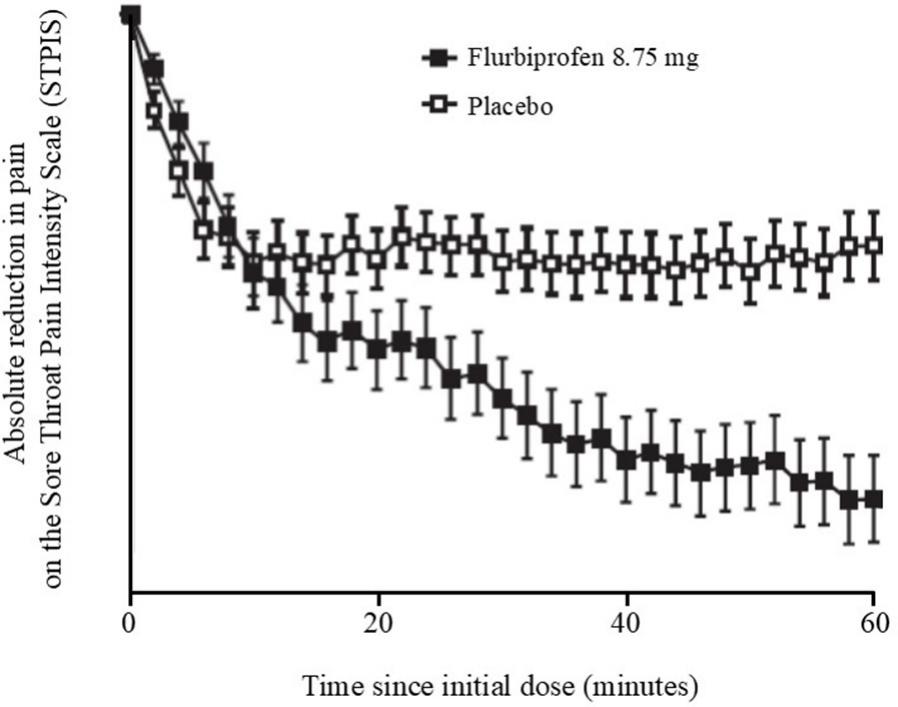
Effects of a single flurbiprofen 8.75 mg lozenge on sore throat pain intensity (measured on the STPIS VAS), adapted from (28). Data shown are mean ± SEM for absolute reduction in pain following a single dose of flurbiprofen or placebo. SEM, standard error of the mean.

A similar approach using 2-minute assessment intervals has also been employed to demonstrate onset of pain relief during the first hour (28). Sore throat pain was measured on the 100 mm STPIS to determine the trajectory of pain reduction of flurbiprofen 8.75 mg lozenge compared to vehicle (placebo) lozenge (Figure 3). The 2-minute assessments facilitated the detection of relief associated with the demulcency attributable to the pharmaceutic vehicle of the lozenge base as well as the pharmacologic effect of the NSAID (i.e., separation of drug from vehicle effects). Flurbiprofen 8.75 mg lozenges were shown to have an immediate demulcent effect for sore throat (measurable within 2 minutes), while the pharmacologic effect of flurbiprofen was evident at 12 minutes, with statistically significant differentiation from the vehicle placebo (*p* = 0.03) beginning at 22 minutes (29). In terms of research methodology, this trial demonstrated that taking one analgesic assessment at 2-minute intervals is feasible, without subject or study nurse fatigue, placebo enhancement, or evidence of regression to the mean. In this trial the 2-minute methodology provided clear evidence of the onset of pharmacologic activity beyond the initial demulcent activity of a topically applied drug. The fast onset of action of the flurbiprofen lozenge (both the pharmaceutical demulcent effect and the co-incident and subsequent pharmacologic effect) led to wide differentiation of drug from placebo, aligning with the conclusion of Moore et al., that fast onset of action correlates with greater overall pain relief (59).

The double-stopwatch (DSW) method, a standard model for measuring the onset of drug activity in other acute pain models, such as post oral surgery and post bunionectomy (63, 64), has also been implemented within the structure of the sore throat pain model (28, 65). Subjects were instructed to depress one stopwatch at the time when they perceived any pain relief and a second stopwatch at the time when they experienced what they considered to be meaningful pain relief. This approach was employed in a randomized, double-blind, placebo-controlled study on 50 and 100 mg doses of celecoxib in patients with acute painful pharyngitis (66). Median values for time to perceptible pain relief were reported as 49 minutes and 61 minutes for 50 mg and 100 mg doses of celecoxib respectively, and 97 minutes for the placebo group. Median time to meaningful relief was not reached for any group within the initial 2-hour in-clinic study period. The DSW method was also employed in a randomized, double-blind study comparing the use of aspirin, acetaminophen, and placebo to treat sore throat pain (67). Both active treatments were significantly differentiated from placebo (*p* < 0.001) in terms of the median time to meaningful pain relief (48.0 minutes for aspirin, 40.4 minutes for acetaminophen, not reached for placebo).

Patients with sore throat also used the DSW method in a randomized, double-blind, placebo-controlled trial of topically applied flurbiprofen 8.75 mg lozenges to assess the times of perceptible and meaningful pain relief (65). This study was also designed to investigate patients’ numeric representations of “perceptible” and “meaningful” pain relief in the DSW method. Patients used an 11-point ordinal rating scale based on the Lasagna Pain Scale (here termed the Sore Throat Scale [STS]) at 5-minute intervals over 1 hour, then at 10-minute intervals for a further 2 hours, with a 100 mm linear scale (STPIS) (65) at 1 hour, 2 hours, 3 hours, and at the timepoint when the second stopwatch was stopped. According to the DSW method, the median time to meaningful relief with flurbiprofen 8.75 mg was 43 minutes. At this timepoint, patients receiving flurbiprofen 8.75 mg reported 42% mean reduction in pain intensity on the STPIS, a rating which indicates “much improvement” and is approaching the criterion for “definite improvement” (53, 65). There was also a clinically meaningful 2.2-point reduction on the 11-point STS for flurbiprofen-treated patients at 45 minutes (the closest prespecified timepoint to the 43 minutes median time to meaningful relief detected by the DSW method). Combining these different methods of detecting pharmacologic activity allowed investigators to not only detect the time of onset of meaningful pain relief but also to determine the level of patient-measured pain intensity at the time of meaningful relief as registered by the DSW method. Concurrently using a PRO with the DSW method thus provided the first direct measurement of the percent change in pain intensity at the time of patient-determined meaningful relief (65). However, because the patient is not reminded at prespecified timepoints to depress a stopwatch if indicative of perceptible or meaningful relief, it appears that the DSW method is not as sensitive as measurements of pain intensity at frequent time intervals for the detection of the onset of demulcent and pharmacologic effects of a topically applied analgesic.

## Duration of analgesia in patients with painful pharyngitis

Sore throat is an acute condition which may last up to several days (68). As such, the sore throat pain model is well suited for examining the duration of analgesic activity over the course of a single dose and after multiple doses over days. The duration of analgesia has been assessed following a single dose of orally administered aspirin 650 mg (19), aspirin 800 mg, aspirin 800 mg with caffeine 64 mg (25), acetaminophen 650 mg or 1,000 mg (19, 31), ibuprofen 200 mg or 400 mg (31, 62), ibuprofen 25 mg lozenge (69), and flurbiprofen 2.5 mg, 5 mg, 8.75 mg, 12.5 mg (21–23, 27, 28, 30, 70). For example, the duration of the single-dose effect of flurbiprofen 8.75 mg lozenge on common throat symptoms (throat pain, swollen throat, difficulty swallowing) was assessed over 6 hours in patients with confirmed pharyngitis (26). Over the 6-hour period, patients taking the flurbiprofen 8.75 mg lozenge experienced significantly greater relief of each of the three PROs compared with patients taking placebo (all *p* < 0.001). In a 24-hour multiple-dose study in patients with relatively severe sore throat pain, throat swelling, and difficulty swallowing, flurbiprofen 8.75 mg lozenges (taken every 3–6 hours as needed; up to 5 lozenges in 24 hours) provided evidence of significantly greater improvements in all three endpoints (40, 61). Flurbiprofen has also been shown to provide significantly greater pain reduction compared with placebo on a per-dose basis from before to 2 hours after each dose taken as needed (70). These multiple-dose studies on flurbiprofen confirmed the safety and efficacy findings of the single-dose trials (71).

## Conclusions and future directions for research

**Figure 4:**
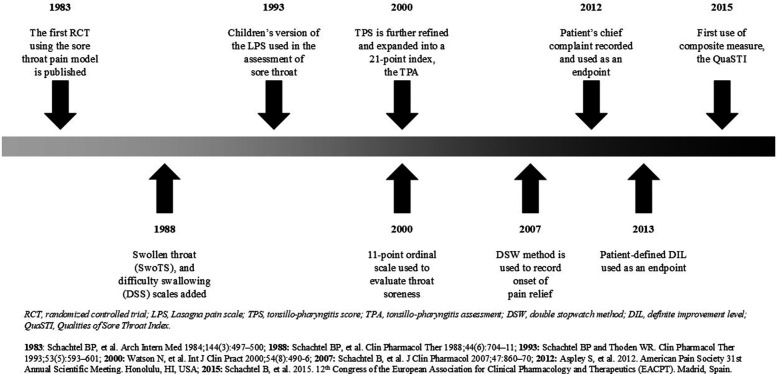
Timeline of key events in the development of the sore throat pain model.

As shown in Figure 4, the sore throat pain model was developed to overcome the methodologic challenges associated with demonstrating analgesic efficacy in individuals with a commonly occurring acute pain condition. Its clinical relevance and utility were evident from research demonstrating the efficacy of topical flurbiprofen compared with placebo, irrespective of the presence of GABHS or GCBHS pharyngitis (72, 73), supporting the provision of effective symptom relief until laboratory confirmation of bacterial infection. This approach is critical given the unreliability of clinicians’ predictions of “strep throat” as a definitive diagnosis of the bacterial (vs. viral) etiology of pharyngitis (72).

Early iterations of the pain model understood the patient-to-patient variability and how simple steps could be taken to ensure consistency in demonstrating analgesic efficacy. For example, in its first iteration, the model featured a number of pre-treatment criteria aimed at ensuring homogeneity in baseline status and quantifying symptom severity as precisely as possible. These criteria included requirements regarding objective features specific to the painful condition, pharyngitis; exclusion criteria based on confounding clinical features of the underlying clinical cause of painful pharyngitis, URTI; and the delineation of the level of severity of the patient-reported pain (20).

The sore throat pain model was enhanced by focusing on the patient, specifically, listening to the words patients use to describe their condition (18). These descriptors served as triggers for creating specific rating scales based on sensory, evaluative, affective, and functional qualities of pain (in particular, the most common terms: difficulty swallowing, the sensation of a swollen throat, sore/painful throat). These patient-defined measurement instruments were shown to be sensitive for assessing change in patient-reported symptoms, while enabling patients to freely report adverse events during the clinical trial.

Focusing on the particular endpoint that was most significant to the patient represented another refinement of the model. For, at its core, the sore throat pain model has a patient-centered orientation. Each patient was permitted to identify the one symptom which was the most bothersome to him/her. This maneuver was achieved in various ways, by inquiring about the patient's “chief complaint” among all his/her symptoms (as clinicians in practice do) and by identifying the patient's most highly rated symptom. In contrast to an investigator-determined endpoint, patients were permitted to determine what mattered most to them and rate changes in this symptom as the primary endpoint analyzed for efficacy in the study. At the same time, the model also incorporated a conventional endpoint regarded as representing meaningful analgesic activity (i.e., at least “moderate relief”). Thus, the model also provided definitive evidence of efficacy based on a standard criterion of therapeutic effectiveness. Finally, the model utilized the patient's own designation of which numeric level (on an 11-point ordinal scale) was indicative of “definite improvement” relative to his/her baseline rating of a particular symptom. In so-doing, the patient defined his/her own criterion of success, with individual results summed and averaged for each treatment group to make comparisons between treatment groups.

Multiple patient-reported descriptors are required to obtain an accurate picture of the impact of sore throat. The STPIS and STS provide an indication of the degree of pain and a means to measure analgesia, and the SwoTS facilitates inclusion of symptomatic swollen throat. The agonizing scale and QuaSTI incorporate more emotional components and the DSS and DTS facilitate patient-reported assessment of the impact of pharyngitis on related functions, such as swallowing. In sum, the methods employed in the sore throat pain model enhance the sensitivity of symptom rating scales and their analyses. With greater assay sensitivity, studies based on the sore throat pain model improve the efficiency of the studies themselves, yielding statistically significant findings with low sample size requirements (25, 52). As such, these methodologic improvements have added to the armamentarium of clinical investigators evaluating the efficacy of new analgesic drugs compared to placebo and compared to standard drugs, differences between dosages of drugs, and analgesic potentiation. Because of its ubiquity as a common acute pain experience, sore throat has also proven to be a valid model for evaluating acute pain in general. Moreover, adaptations of these methods can be applied to clinical pharmacology assays for other clinical conditions (e.g., nasal congestion, sinus pain, tension-type headache, migraine headache, cough, post-operative pain, abdominal discomfort, arthritic pain and stiffness, low back pain, etc.). In so-doing, the development and evaluation of future medicines can go beyond the requirements of a single conventional endpoint and examine other, patient-centered benefits.
